# Multi-sector determinants of implementation and sustainment for non-specialist treatment of depression and post-traumatic stress disorder in Kenya: a concept mapping study

**DOI:** 10.1186/s43058-025-00744-7

**Published:** 2025-05-07

**Authors:** Erika L. Crable, Susan M. Meffert, Ryan G. Kenneally, Linnet Ongeri, David Bukusi, Rachel L. Burger, Grace Rota, Ammon Otieno, Raymond Rotai, Muthoni Mathai, Gregory A. Aarons

**Affiliations:** 1 Department of Psychiatry, University of California San Diego, La Jolla, CA USA; 2 Herbert Wertheim School of Public Health and Human Longevity Science, University of California San Diego, La Jolla, CA USA; 3 ACTRI Dissemination and Implementation Science Center, University of California San Diego, La Jolla, CA USA; 4 Child and Adolescent Services Research Center, San Diego, CA USA; 5 Department of Psychiatry, University of California San Francisco, San Francisco, CA USA; 6 Centre for Clinical Research, Kenya Medical Research Institute (KEMRI), Nairobi, Kenya; 7 Department of Psychiatry, University of Nairobi, Nairobi, Kenya; 8 Department of Obstetrics, Gynecology and Reproductive Sciences, University of California San Francisco, San Francisco, CA USA; 9 University of Nairobi, Nairobi, Kenya

**Keywords:** Concept mapping, EPIS Framework, Implementation science, Policy, Non-specialist workforce, Mental health

## Abstract

**Background:**

The global shortage of trained mental health workers disproportionately impacts mental health care access in low- and middle-income countries. In Kenya, effective strategies are needed to scale-up the workforce to meet the demand for depression and post-traumatic stress disorder treatment. Task-shifting – delegating specific tasks to non-specialist workers – is one workforce expansion approach. However, non-specialist workers remain underutilized in Kenya due to a paucity of research on how to scale-up and sustain such service models.

**Methods:**

Purposive sampling was used to recruit experts from policy, healthcare practice, research, and mental health advocacy roles in Kenya (*N* = 30). Participants completed concept mapping activities to explore factors likely to facilitate or hinder a collaborative Ministry of Health-researcher training of the mental health non-specialist workforce. Participants brainstormed 71 statements describing determinants and implementation strategies, sorted and rated the importance and changeability of each. Multidimensional scaling and hierarchical cluster analysis quantified relationships between statements. The Exploration, Preparation, Implementation, and Sustainment (EPIS) framework guided cluster interpretation activities.

**Results:**

Twelve determinant clusters were identified: 1) Current workforce characteristics, 2) Exploration considerations, 3) Preparation considerations, 4) Sustainment considerations, 5) Inner context implementation processes and tools, 6) Local capacity and partnerships, 7) Financing for community health teams, 8) Outer context resource allocation/policy into action, 9) Workforce characteristics to enhance during implementation, 10) Workforce implementation strategies, 11) Cross-level workforce strategies, and 12) Training and education recommendations. Cluster 8 was rated the most important and changeable.

**Conclusion:**

Concept mapping offers a rapid, community-engaged approach for identifying determinants and implementation strategies to address workforce shortages. Organizing results by EPIS phases can help prioritize strategy deployment to achieve implementation goals. Scale-up and sustainment of the non-specialist workforce in Kenya requires formal partnerships between the Ministry of Health and community health worker teams to distribute financial resources and collaboratively standardize training curriculum.

**Supplementary Information:**

The online version contains supplementary material available at 10.1186/s43058-025-00744-7.

Contributions to the literature

- This study leveraged expertise frompolicy, healthcare practice, research stakeholders, and mental health advocates to identify phased implementation strategies required for successful non-specialist a workforce intervention in Kenya.
- Concept mapping clusters represent determinants that are expected to influence nationwide policy implementation decisions.
- Formal partnerships between government and community health worker teams are needed to maintain a national commitment to increasing mental healthcare access and increasing the distribution of new resources to support the non-specialist workforce.
- Organizing concept mapping determinant cluster results through an implementation science framework helps prioritize the timing and delivery of implementation strategies in multi-level policy implementation efforts.

## Background

There is a growing and unmet need for mental healthcare globally. The Global Burden of Disease Study 2021 identified substantial increases in rates of disability adjusted life-years and years lived with disability for anxiety and depressive disorders between 2010 and 2021 [1]. Within the first year of the COVID-19 pandemic, globally measured rates of depression increased 25% [2]. Depression is expected to be a leading cause of burden of disease by 2030 [3]. The shortage of trained mental health workers is a pressing global health challenge disproportionately impacting low- and middle-income countries (LMICs) [4]. LMICs have an estimated shortage of 1.18 million mental health workers [5]. Effective strategies to scale up and sustain the mental health workforce are urgently needed to address common conditions including depression and post-traumatic stress disorder (PTSD).

Major depressive disorder and PTSD are two of the most prevalent mental disorders in Kenyan primary care populations [6–8]. Prior research investigating access to mental health services across Kenya’s counties found that in some counties, only 1.7% of individuals with a need for treatment received services [9]. To address this substantial treatment gap, in 2021, the Kenyan government launched the Kenya Mental Health Action Plan aimed at improving mental health system leadership and governance, increasing access to mental healthcare by integrating services with primary care, and strengthening mental health systems through investments in the workforce size and available training supports [10]. Mobilizing the local non-specialist mental health workforce can help address the existing significant mental health treatment gap in Kenya. Non-specialist health workers include any type of health workers (e.g., doctors, nurses, community and lay health workers) who are not specialists of mental healthcare, but who may have had some training for common conditions [11]. Non-specialist workforce interventions are effective for increasing access to treatment, reducing symptoms of PTSD, supporting recovery from depression and anxiety [11], and promoting culturally responsive care [12]. Yet the non-specialist workforce remains underutilized, in part due to a paucity of implementation research demonstrating strategies to effectively scale-up such interventions. [8]. Most studies of non-specialist services for depression and PTSD have focused primarily on behavioral interventions [13–15]. However, there is a need for both psychotherapeutic and pharmacological treatments for these mental health disorders [16–22].

This study applied concept mapping methods through an implementation science lens to identify implementation and sustainment determinants (i.e., barriers and facilitators) and cross-level alignment promoting factors to support non-specialist workforce scale up in Kenya’s public sector mental healthcare system to address depression and PTSD. To confront this research-practice knowledge gap, we conducted a concept mapping study [23] with diverse stakeholders from Kenya’s mental health system including policymakers, advocates, and researchers. We aimed to identify critical determinants and mechanisms of ongoing efforts to collaborate with the Ministry of Health, public health facilities and hospitals in training the non-specialist workforce in mental healthcare within Kenya’s public health system.

## Methods

Concept mapping activities were conducted within the context of an effectiveness-implementation Hybrid type 1 larger parent grant that is a Sequential, Multiple, Assignment Randomized Trial (SMART) of two evidence-based treatments for depression and PTSD (the SMART-DAPPER study) in Kenya. The SMART-DAPPER parent trial randomized 2,710 patients in outpatient clinics at Kisumu County Hospital and other Kisumu County Ministry of Health facilities to receive either fluoxetine or Interpersonal Therapy (IPT) provided by non- mental health specialists (e.g., nurses, community health workers, teachers, church leaders, women’s group leaders, village chiefs/elders, traditional healers). The parent trial tested the effectiveness of and treatment fidelity to each evidence-based practice. The study team trained nurses and clinical officers to deliver fluoxetine, and non-specialist therapists participated in an IPT training course. Both training courses were delivered over multiple days and included subsequent proctored treatment sessions with multiple practice participants and supportive supervision [8]. Given the focus on non-specialists’ role in delivering IPT and fluoxetine, the study also aimed to investigate potential strategies to scale-up non-specialist mental healthcare delivery in Kenya in the event that non-specialist delivered IPT and fluoxetine was highly effective. The parent trial convened an Implementation Resource Team (IRT) [24, 25] comprised of cross-sector key informants to shepherd implementation activities across study aims. All IRT participants were then asked to participate in the concept mapping study to provide insights about how to leverage and sustain a non-specialist workforce to deliver mental healthcare. Additional details of the SMART-DAPPER parent study are described elsewhere [8]. This research was approved by the University of California San Francisco Institutional Review Board.

Concept mapping is a mixed methods (qualitative and quantitative) approach to meaningfully engage invested actors, collaborators, and end users in an implementation effort in the research process [23, 26–28]. Concept mapping can be used to foster communication about implementation efforts between actors across contexts, and incorporate their diverse perspectives, knowledge, and positionalities into implementation and sustainment planning. First, individual and group brainstorming takes place to enumerate potential determinants (i.e., implementation barriers and facilitators). Next, sorting activities and analyses are used to generate a conceptual framework and shared understanding relevant for the implementation context and clinical interventions. These initial steps use participant language to identify relevant determinants and potential implementation strategies [23]. Next, each statement is rated on its importance and changeability. Then quantitative methods are used to identify key determinants likely to affect scale-up depression and PTSD treatment.

### Participants

The parent trial (SMART DAPPER) employed purposive sampling [29] to recruit participants with professional or lived experience roles in diverse sectors (i.e., policy, healthcare practice, research, and mental health advocacy) to inform the implementation of non-specialist delivered care for depression and PTSD in Kenya. Policy actors included Kenyan officials from national and county health entities. Healthcare practice perspectives included healthcare workers and leaders from health facilities and non-governmental organizations. University-affiliated researchers and consultants were included in the research perspective, and mental health advocates were recruited from non-governmental and other organizations involved in mental healthcare promotion including some individuals who were receiving IPT and fluoxetine. This group was identified as the IRT – a resource to provide ongoing key informant insights and feedback to the parent trial. The research team invited all IRT members to participate in concept mapping activities.

### Theory

The Exploration, Preparation, Implementation, Sustainment (EPIS) framework guided data collection and interpretation activities. The EPIS framework is both a process and determinant framework that describes how determinants and relationships between entities in the local implementation context (inner context) and the multilevel sociopolitical and service delivery environments (outer context) can change across the four phases of implementation and influence implementation outcomes and sustainment of evidence-based practices [30, 31]. EPIS “bridging factors” represent relational ties, formal arrangements, and other processes for supporting outer and inner context alignment for implementation success and long-term sustainment [32]. Bridging factors can also include structures or ways of exchanging financial or political capital to promote policy transfer, ensuring national health priorities are pursued by local governments and adhered to in public sector clinics [33]. The present study documented exploration and preparation phase concept mapping activities that occurred, and plans for future implementation and sustainment activities. During the exploration phase, invested stakeholders across outer and inner contexts defined the need to improve mental health services, and agreed to focus on enhancing the non-specialty mental health workforce to augment depression and post-traumatic stress disorder service delivery. During the preparation phase, cross-context representing participants (i.e., IRT members) considered determinants (i.e., barriers, facilitators) and potential implementation strategies to expand the non-specialty mental health workforce to provide treatment for depression and PTSD. The implementation and sustainment phases were not observed, but concept mapping activities revealed key determinants and implementation strategies relevant to progress into these phases.

### Data collection for concept mapping

**Fig. 1:**
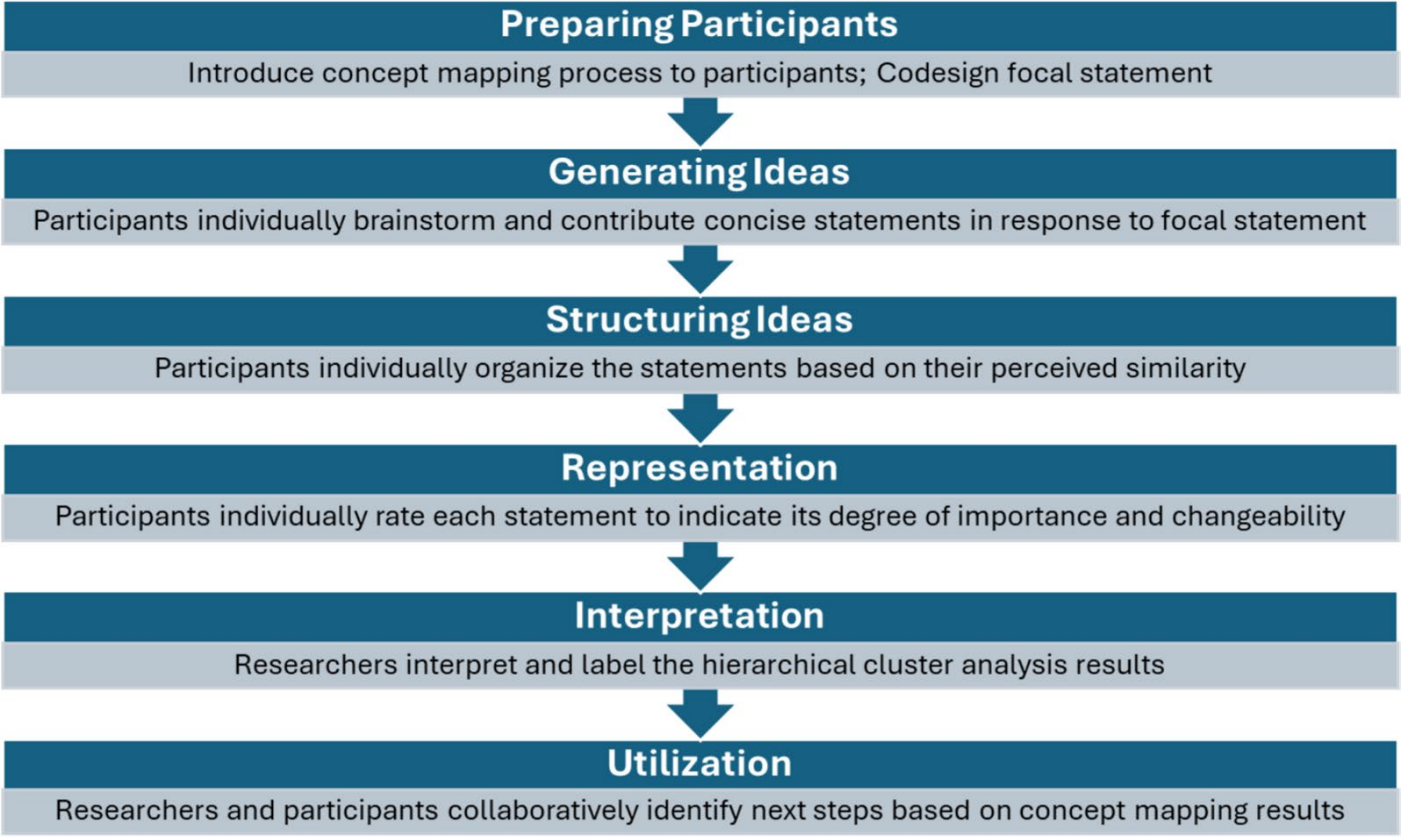
Concept mapping stages

Concept mapping activities occurred in six stages: (1) preparing participants, (2) generating ideas, (3) structuring ideas, (4) representation, (5) interpretation, and (6) utilization (Fig. 1) [23, 28]. During the preparation stage, IRT members met over a video call to discuss the unmet need for depression and PTSD care in Kisumu, Kenya. A member of the research team introduced the concept mapping process to IRT members and facilitated the group in generating a focal statement for concept mapping process: *“What factors will facilitate or hinder a collaborative Ministry of Health-SMART DAPPER training of Kenyan mental health non-specialist workforce for scale up and sustainment of public sector mental healthcare?”* IRT members were instructed to respond to the focal statement by generating concise statements regarding potential barriers and facilitators to training a non-specialist workforce to scale up and sustain public sector mental healthcare. Participants generated 40 unique statements during the initial call.

IRT members were asked to engage in additional individual idea generation (i.e., brainstorming) and structuring stage activities (i.e., sorting statements) via the web-based concept mapping platform, GroupWisdom.^34^ Participants received an emailed invitation to the platform where they encountered web-based prompts to guide them through the process. The study team used a web-based platform to conduct concept mapping activities (instead of in-person meetings) given the geographic dispersion of participants, the desire to promote asynchronous and anonymous responses to the focal statement, and the need for social distancing during the COVID-19 pandemic. IRT members were asked to respond to five questions about their knowledge of evidence-based practices for mental health treatment, educational experiences regarding depression and PTSD topics, their amount of previous experience participating in collaborative efforts to improve mental healthcare or advocating for the rights of people living will mental illness, and their perceived ability to enact changes to Kenya’s public-sector mental healthcare delivery system. Participants were then instructed to review the list of 40 brainstorming statements and contribute any additional relevant ideas in response to the focal statement. Participants could enter as many statements as they desired, and view statements generated by other participants. All statements were anonymously viewable to participants. The research team reviewed the list to remove duplicative ideas, combine similar statements, and ensure that only one concise idea was represented in each statement. A total of 71 unique statements were generated.

During the structuring the GroupWisdom platform enabled participants to electronically sort all statements into different ‘piles’ based on their perceived similarity, yielding as many piles as each participant deemed meaningful. Participants could name the piles if desired. Participants also rated each statement separately to indicate its degree of importance and changeability (e.g., how feasible or possible it is address or alter a barrier to implementation) using a scale of one to five. Participants first rated how important each determinant statement was to the implementation of training the Kenyan mental health non-specialist workforce for scale up and sustainment of public sector mental healthcare. Then participants rated how changeable each determinant was to address during implementation efforts.

### Generating the concept map

Once data collection was completed, concept mapping procedures progressed to the representation stage. A similarity matrix for each participant was generated to indicate where each statement was, or was not, sorted together with other statements. Next, we conducted multidimensional scaling using the matrix data for all participants to place statements on a two-dimensional point map. Hierarchical cluster analysis was used to develop a cluster map depicting which statements cluster together into relatively mutually exclusive conceptual clusters of statements [34]. Hierarchical cluster analysis is an algorithm that is useful for identifying patterns in data, such as statements that are conceptually similar to each other and meaningfully distinct from other statements generated by participants. We used a hierarchical cluster analysis algorithm to identify patterns of statements that participants frequently sorted together, and then represented those clustered statements as visually closer together on the point and cluster maps. To conduct the hierarchical cluster analysis, the research team reviewed the 71 participant generated statements and reviewed the point and cluster maps to estimate the optimal number of clusters that would practically make sense and represent the statement data generated by participants. We estimated a series of models, each with an increasing number of potential clusters ranging from 10 to 16 clusters. To select the final model, the research team considered how well the number of clusters could be interpreted as meaningfully distinct themes and considered the model’s compatibility with our initial estimate. Average bridging values and spanning values for statements were calculated to describe the relationship between statements across clusters and to confirm that clusters were conceptually distinct. The research team interpreted cluster themes to assign cluster labels. Cluster labels were cross-walked to EPIS constructs [30, 31] (i.e., outer context, inner context, bridging factors, innovation factors) to enhance interpretability and use in guiding future global mental health implementation efforts.

Finally, we conducted pattern matching analysis to compare average ratings for the importance and changeability of each statement aggregated to the cluster level and measured the average correlation between these dimensions. Additionally, each statement’s importance and changeability score was plotted on a graph and divided into four quadrants using the mean of importance and changeability scores to model a ‘Go-Zones’ plot [23, 28]. Statements in quadrant 4 (i.e., upper right Go-Zone) have above average values for both importance and changeability and represent the focal statements that participants prioritized for addressing in future implementation efforts. A presentation summarizing the concept mapping methods and process was shared with IRT members.

## Results

### Sample characteristics

Thirty IRT members participated in concept mapping activities including: responding to participant experience questions (*n *= 30), brainstorming (*n* = 30 and sorting statements (*n* = 16), and rating importance and changeability (*n* = 15; Table 1). Most IRT members (70%) rated themselves as knowledgeable to very knowledgeable about evidence-based practices for mental health treatment. IRT members self-reported having formal (60.0%) and informal (56.7%) educational experiences with depression and PTSD, and 73.4% had “moderate” to “a lot” of experience advocating for the rights of people living with mental illness or advocating for mental healthcare services. Almost all members (93.3%) had prior experience participating in other collaborative, multisector efforts to improve mental healthcare. Most members (63.4%) said they had a “great extent” of ability to enact change in the delivery of evidence-based mental healthcare in Kenya’s public sector. Notably, none of the participants reported having no ability to enact change.

**Table 1:** Implementation Resource Team participant characteristics

**Sample characteristics**	**Sample size** *N* = 30 (%)
Professional affiliation	
Government agency policy actors	12 (40.0)
Healthcare practitioners	8 (26.7)
Academic researchers	2 (6.6)
Mental health advocates	8 (26.7)
Which kinds of educational experiences have you had regarding depression and PTSD (select all that apply)?	
Formal training	18 (60.0)
Informal training	17 (56.7)
Lived experience	12 (40.0)
None of the above	1 (3.3)
How would you rate your knowledge of evidence-based practices for mental health treatment (i.e., services that have been scientifically shown to be effective in improving mental health outcomes)?	
Very knowledgeable	5 (16.7)
Knowledgeable	16 (53.3)
Somewhat knowledgeable	6 (20.0)
Not knowledgeable	3 (10.0)
How much experience do you have participating in collaborative, multisector (e.g., working with governmental, non-governmental, and provider organizations) efforts to improve mental healthcare?	
A lot of experience	9 (30.0)
Moderate experience	14 (46.7)
Some experience	5 (16.6)
No experience	2 (6.7)
How much experience do you have advocating for the rights of people with mental illness or advocating for mental healthcare services?	
A lot of experience	11 (36.7)
Moderate experience	11 (36.7)
Some experience	7 (23.3)
No experience	1 (3.3)
To what extent do you feel that you can enact change in the delivery of evidence-based mental healthcare for depression and PTSD in Kenya’s public sector?	
A great extent	19 (63.4)
Somewhat	7 (23.3)
Very little	4 (13.3)
Not at all	0 (0)

### Final concept map

**Fig. 2:**
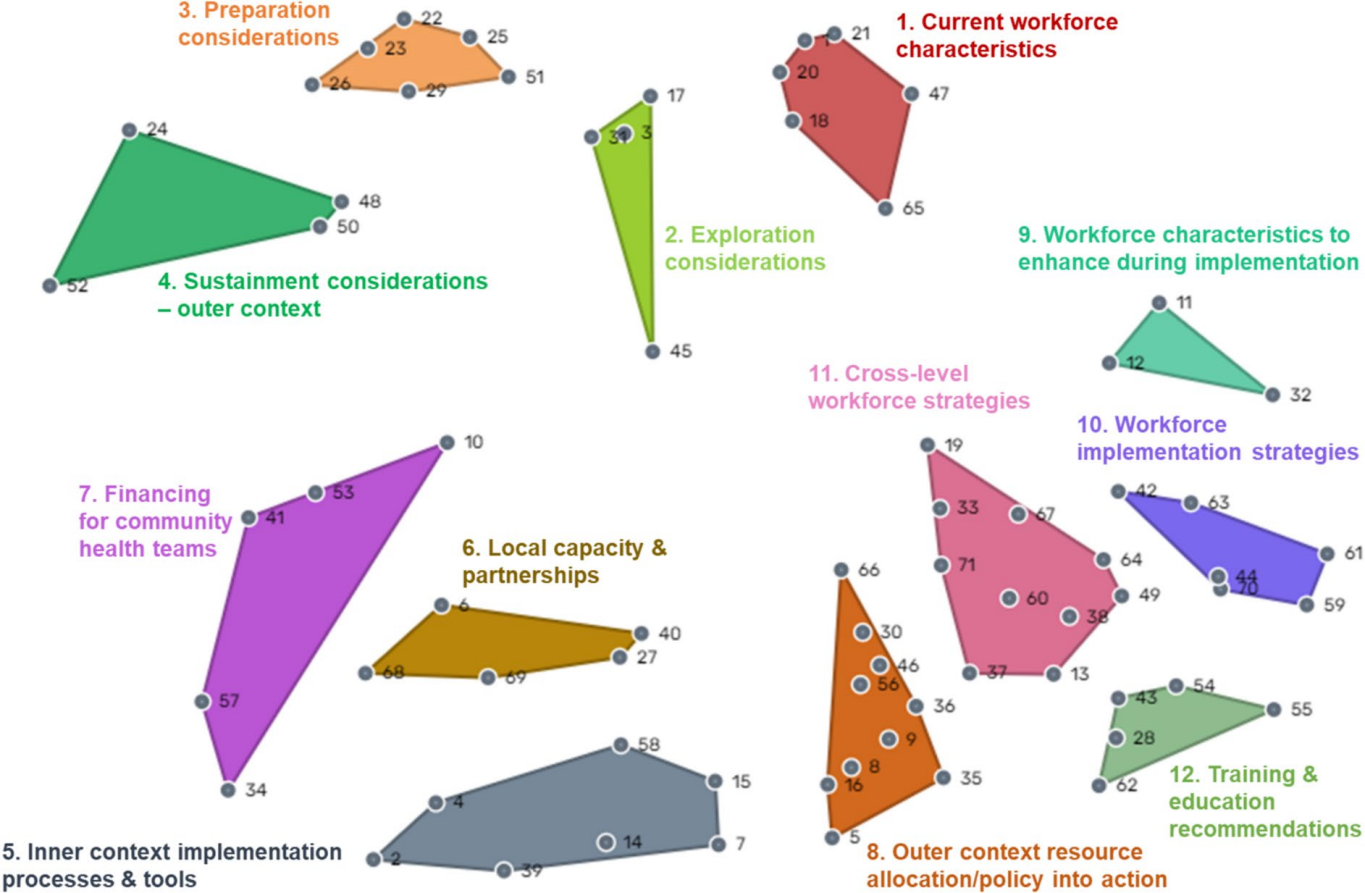
Cluster and point map of factors expected to influence training of Kenyan mental health non-specialist workforce. Note: Colored shapes and labels represent thematically distinct clusters. Numbered points in grey represent the 72 discrete statements that participants generated and sorted during the concept mapping process (numbered statements are listed in Appendix 1). Spatial distance between statements illustrates how frequently statements were sorted into the same pile as other statements

A 12-cluster model was selected as the final model. Figure 2 displays the final point and cluster map (i.e., ‘concept map’) illustrating relationships between the 71 statements grouped into 12 clusters. The clusters represented 1) Current workforce characteristics, 2) Exploration considerations, 3) Preparation considerations, 4) Sustainment considerations, 5) Inner context implementation processes and tools, 6) Local capacity and partnerships, 7) Financing for community health teams (referred to as ‘village health teams’ in the statement list), 8) Outer context resource allocation/policy into action, 9) Workforce characteristics to enhance during implementation, 10) Workforce implementation strategies, 11) Cross-level workforce strategies, and 12) Training and Education. Proximity of mapped clusters, and bridging values (i.e., the degree to which statements within one cluster could “bridge” or be similar to statements in nearby clusters) revealed some thematic similarities between the clusters. For example, strong bridging values were measured between clusters 4 (i.e., Sustainment considerations) and 7 (i.e., Financing for community health teams) (0.56–0.69), indicating strong conceptual linkage between the clusters. Statements in cluster 4 describe low reimbursement rates and staff turnover during implementation as factors affecting sustainment of non-specialist workforce training efforts. Neighboring cluster 7 describes the need to specifically finance community health worker teams for implementation success.

Clusters 1–4, and 8 represent EPIS outer context determinants expected to impact the training efforts for the Kenyan mental health non-specialist workforce. Participants identified current workforce characteristics (cluster 1) including turnover rates, a lack of clear roles and responsibilities, and resistance to change as general concerns. Participants generated ideas for supporting training efforts across implementation phases. EPIS Exploration considerations (cluster 2) include clarifying Ministry of Health responsibilities and assessing current mental health service quality, and client treatment seeking behaviors. EPIS Preparation considerations (cluster 3) focus on generating awareness and support for SMART DAPPER and considering potential barriers to scaling up the workforce. EPIS Sustainment considerations (cluster 4) describe ongoing factors likely to influence the size and quality of the non-specialist workforce in public sector mental healthcare such as insufficient service reimbursement rates. Clusters 5, 9, and 12 address inner context considerations. Cluster 5 statements focus on inner context implementation processes and tools including timely communication, effective supervision for non-specialist providers, patient follow-up and navigation services. Cluster 9 describes specific workforce characteristics to enhance during implementation (i.e., outcomes of implementation strategies), while cluster 12 proposes workforce training and education activities.

Themes from clusters 6, 7, 10, 11 represent potential EPIS bridging factors to align outer and inner context entities in a common goal of scaling up and sustaining the non-specialist mental health workforce. Cluster 6 focuses on how community health worker teams (inner context) and Ministry of Health (outer context) entities can support local capacity and partnerships for implementation success. Cluster 7 highlights the need for dedicated financing for community health worker teams. While clusters 1, 9, and 12 described workforce contextual determinants likely to impact training efforts, clusters 10 and 11 describe potential strategies for non-specialist workforce training success. Cluster 10 describes implementation strategies related to capacity building across levels of the health system and continuous medical education. Cluster 11 describes ways to integrate non-specialists into existing systems including the development of a training curriculum and certification process.

**Fig. 3:**
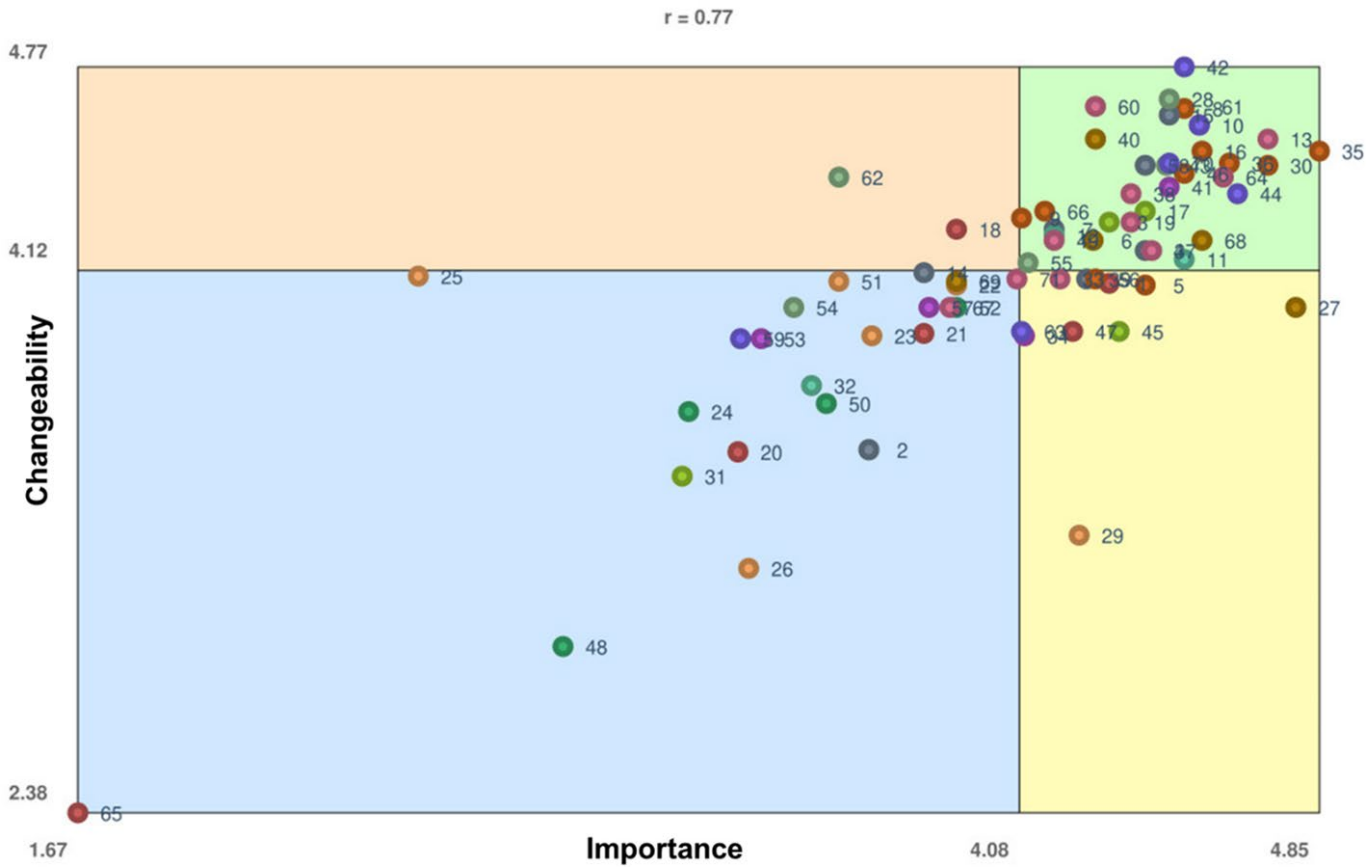
Go-Zone map. The Go-Zone map shows the mean participant rating for each statement brainstormed and rated in the concept mapping study. The X-axis represents ratings of how important each item is, and the Y-axis represents ratings of how changeable each item is as perceived by concept mapping participants. The colors of each point illustrate which cluster the statements belong to (see Fig. 2)

Table 2 displays all statements, grouped by cluster labels, and with individual importance and changeability ratings, and Go-Zone quadrant positions. There was a strong correlation (*r* = 0.77) between importance and changeability ratings indicating that most statements were mapped in Go-Zone quadrant 1 (high importance and changeability; Fig. 3, Supplemental Material). Similarly, the Pearson product-moment correlation (*r* = 0.93) measured a high level of predictable alignment between average cluster ratings of importance and changeability ratings. Thirty-five statements (49.3%) were mapped in the Go-Zone for their high importance and changeability ratings. Average cluster importance and changeable ratings revealed that IRT members perceived outer context resource allocation (cluster 8) to be the most important (4.46) and changeable (4.37) area of focus.

**Table 2:** Summary of 71 factors expected to influence training of Kenyan mental health non-specialist workforce, organized by cluster with mean importance and changeability ratings and Go-Zone quadrant

Cluster	Numbered statement ordered by cluster and highest combined importance and changeability score	Importance	Changeability	Quadrant
**1** Current workforce characteristics	(20) Resistance from professionals who see task shifting as poor medicine for poor people	3.3571	3.5385	1
	(21) High turn-over of trained workers	3.8333	3.9167	1
	(65) Do not introduce a new mental health cadre	1.6667	2.3846	1
	(1) High staff turnover negatively affects such collaborative efforts	4.3077	4.0769	2
	(47) Unmotivated workforce	4.2143	3.9231	2
	(18) Lack of clear roles and responsibilities	3.9167	4.25	3
**2** Exploration considerations	(31) Ministry of Health view that mental health non-specialist workforce are not a reliable cadre of personnel	3.2143	3.4615	1
	(45) Quality of health services	4.3333	3.9231	2
	(63) Engage professionals from different fields as non-specialist providers	4.0833	3.9231	2
	(3) Lack of ownership and responsibility from the Ministry of Health officials	4.3077	4.2727	4
	(17) Limited knowledge about mental illness prevents individuals from recognizing mental illness and seeking treatment	4.4	4.3077	4
**3** Preparation considerations	(22) Few satellite research centers to support SMART-DAPPER	3.9167	4.0714	1
	(23) Low awareness about the SMART-DAPPER research	3.7	3.9091	1
	(25) Negative attitudes toward SMART-DAPPER research	2.5385	4.1	1
	(26) Poverty	3.3846	3.1667	1
	(51) Scalability challenges	3.6154	4.0833	1
	(29) Misperceptions and belief systems about the causes of mental illnesses	4.2308	3.2727	2
**4** Sustainment considerations—outer context	(24) Insufficient participant reimbursement rates	3.2308	3.6667	1
	(48) Government jobs are sought after	2.9091	2.9167	1
	(50) Consider stakeholder turnover during implementation	3.5833	3.6923	1
	(52) Sustainment challenges	3.9167	4	1
**5** Inner context implementation processes and tools	(2) Provision of electronic navigation	3.6923	3.5455	1
	(14) Provide in-person navigation	3.8333	4.1111	1
	(39) Addition to EMS	4.25	4.0909	2
	(4) Timely communication, both formal and informal	4.4	4.1818	4
	(7) Effective treatment of mentally ill patient	4.1667	4.25	4
	(58) Supervision for non-specialized providers	4.4	4.4545	4
	(15) Follow-up with participants at the community level to better understand access to care issues	4.4615	4.6154	4
**6** Local capacity and partnerships	(69) Have a village health team (local people within the community)	3.9167	4.0833	1
	(27) Identify partners with shared interests	4.7857	4	2
	(6) Community mental health service seeking	4.2667	4.2143	4
	(40) Initiate sensitization sessions with stakeholders about mental health service delivery	4.2727	4.5385	4
	(68) Ministry supported community health workers	4.5455	4.2143	4
**7** Financing for community health teams	(53) Payment for village health teams	3.4167	3.9	1
	(57) Engage village health team members to provide care	3.8462	4	1
	(34) Facilitate airtime reimbursement	4.0909	3.9091	2
	(41) Allocated financial resources (budgeted funds for this scale-up process)	4.4615	4.3846	4
	(10) Political will/support	4.5385	4.5833	4
**8** Outer context resource allocations/policy into action	(5) Timely mental health assessment and treatment	4.4	4.0714	2
	(56) Add mental health education to the village health team manual	4.2727	4.0909	2
	(9) Availability of medicines	4.0833	4.2857	4
	(16) Survey questions should yield appropriate information about participant needs	4.5455	4.5	4
	(30) Engage government (e.g., Ministry of Health, Ministry of Education, etc.) leadership in initial planning activities	4.7143	4.4545	4
	(35) Availability of mental health services in all levels of healthcare delivery points	4.8462	4.5	4
	(36) Government and leadership policies that promote sustainability	4.6154	4.4615	4
	(46) The county's commitment to improving mental healthcare	4.5	4.4286	4
	(66) Use existing community health strategy	4.1429	4.3077	4
	(8) Availability of drugs, equipment and commodity needed for mental health treatment	4.5	4.6364	4
**9** Workforce characteristics to enhance during implementation	(32) Non-specialist workforce should play a major role	3.5455	3.75	1
	(11) Workforce capacity—staffing	4.5	4.1538	4
	(12) Workforce qualifications, skill level	4.1667	4.2308	4
**10** Workforce implementation strategies	(59) Train psychologists to supervise community health workers	3.3636	3.9	1
	(42) Capacity building of healthcare workers at all levels in management of mental health	4.5	4.7692	4
	(44) Offering continuous medical education to the providers	4.6364	4.3636	4
	(61) Train nurses and clinical officers on IPT	4.5385	4.5833	4
	(70) Involve educational institutions	4.4615	4.4615	4
**11** Cross level workforce strategies	(67) Integrate non-specialists into government service system	3.9	4	1
	(71) Non-specialists help address stigma	4.0714	4.0909	1
	(33) Availability of human capital in the health products and technology field	4.1818	4.0909	2
	(13) Collaboration between the Ministry of Health and mental health non-specialist workforce in implementing training program	4.7143	4.5385	4
	(19) Clear definition for identifying the mental health non-specialist workforce	4.3636	4.2727	4
	(37) Government and leadership policies that promote continuous mentorship/training for the mental health non-specialist workforce	4.4167	4.1818	4
	(38) Collaborative, public–private partnerships to build capacity for non-specialist workforce	4.3636	4.3636	4
	(49) Identify who will be trained (e.g., police, teachers)	4.1667	4.2143	4
	(60) Have a targeted mental health curriculum	4.2727	4.6429	4
	(64) Make the existing mental health cadre more competent	4.6	4.4167	4
**12** Training and education recommendations	(54) Training manual should be brief	3.5	4	1
	(62) Provide a certificate for IPT	3.6154	4.4167	3
	(43) Expansion of mental healthcare from hospital to learning institutions	4.4545	4.4545	4
	(55) Training manual should be modular	4.1	4.1429	4
	(28) Enhance psychoeducation	4.4615	4.6667	4

## Discussion

This study used concept mapping activities to address a persistent knowledge gap about how to effectively plan for and engage multi-sector stakeholders in efforts to scale-up the non-specialist mental health workforce.^8^ Concept mapping activities represented a rapid, virtual and community engaged approach to leverage the combined knowledge and expertise of stakeholders from policy, healthcare practice, research, and mental health advocacy perspectives. Results specifically revealed factors that facilitate or hinder (i.e., determinants) a collaborative effort between researchers and the Kenya Ministry of Health to train, employ, and sustain the mental health non-specialist workforce’s role in delivering public sector mental health services for depression and PTSD.

The main determinants and mechanisms identified in the concept mapping results represent key domains in the EPIS framework (i.e., outer context, inner context, bridging factors). Outer context (e.g., Ministry of Health and country-wide efforts) and inner context (e.g., local healthcare delivery settings) determinants highlighted different aspects of how workforce characteristics, turnover rates, current skill level and training and education needs are important targets for implementation strategies. Participants also emphasized the need for bridging factor type implementation strategies and supports that can align outer and inner contexts^32^ including formal partnerships between the Ministry of Health and local community health worker teams to distribute necessary financial resources (e.g., government finances to pay the local workforce) and collaboratively refine and standardize the mental health training curriculum. Such implementation strategies may be realized as the Kenyan government recently announced plans to hire 103,000 community health workers that were expected to be trained by the United States Agency for International Development [35]. Concurrent workforce enhancement efforts should coordinate implementation strategies and lessons learned to maximize impact. Future research is needed to independently evaluate government sponsored workforce initiatives.

The outer context emphasis on enhancing government’s formal participation in workforce scale-up efforts through greater resource allocation (e.g., increased availability of medicines, equipment) and policy into action efforts (e.g., leadership engagement throughout planning activities, maintaining a national commitment to improving healthcare, designing policies with sustainment in mind) was identified as the most necessary cluster of implementation strategies to promote widespread scale up of the non-specialists workforce for depression and PTSD service delivery. Prior systematic reviews highlight the importance of obtaining formal engagement from government entities to enhance acceptability and credibility of a non-specialist workforce [36, 37], but have not clarified the specific types of supports a Ministry of Health or other entity should offer. By including policymaker perspectives in the concept mapping exercise, the current study offers insights about what specific outer context government agency supports are needed and realistically available. Importantly, this project included policymaker perspectives while retaining the insights of nonspecialist providers, specialists, and individuals with lived experience. Including a diverse perspectives is critical to ensuring the feasibility and appropriateness of factors and implementation strategies identified in the concept map.

Participants rated all of the statements for their perceived importance and changeability which helps stakeholders to prioritize which cross-context implementation strategies will be needed over time to achieve implementation goals. Viewed through the lens of our guiding EPIS framework [31], a determinants’ importance and potential for changeability informs whether to pursue that factor during Exploration (e.g., identifying determinants and mechanisms related to potential evidence-based practice needs; cluster 2), Preparation (e.g., matching implementation strategies to determinants; cluster 3), Implementation (i.e., testing the implementation strategies and evaluating implementation outcomes; clusters 1, 5–12), and Sustainment (cluster 4). Successful planning for Sustainment is critical to institutionalizing evidence-based interventions that yield continued positive impacts on provider behaviors, patient engagement, patient outcomes (inner context), and ideally population health, policies and funding to support widespread access to evidenced-based depression and PTSD care (i.e., outer context).

Notably, Exploration, Preparation, and Sustainment Consideration focused clusters had lower importance and changeability ratings compared to clusters that focused on Implementation phase issues. Sustainment Considerations was ranked as the least important and least changeable cluster. This is consistent with prior research noting that sustainment strategies are undervalued during active implementation [38–40]. However, planning for sustainment from the beginning can reduce time and financial waste while increasing the public health impact of an implementation effort [41]. Ongoing workforce enhancement efforts will need to identify ways to address workforce reimbursement rates, turnover, and other sustainment challenges identified by participants. For example, compensation can include clear salary scales for non-specialist workers and compensation for travel and participation in workforce training activities [36, 42]. Compensating supervisors of non-specialists may also be an important consideration [42]. Additionally, worker turnover can be significantly reduced when providers perceive the care being delivered as meeting the needs of patients (i.e., strong when there is a strong fit between providers’ values and the service model), and when providers are supported with ongoing training and coaching implementation strategies [43].

Finally, concept mapping activities revealed several existing workforce characteristics that will need to be addressed by Implementation phase activities to scale up the mental health non-specialist workforce to deliver depression and PTSD services. For example, participants highlighted lack of clarity regarding non-specialist roles and responsibilities as a highly important and changeable factor for successful scale up. Poor role clarity is common amongst non-specialist workers [44–46], and can promote competition amongst peers and burnout.^47^ Supervisor mentoring and training programs tailored to non-specialist provider responsibilities can promote a clear mission and job expectations and increase job satisfaction [47, 48]. In recognition of these challenges, participants described the need for workforce implementation strategies including training supervisors to oversee non-specialists, involving educational institutions in training efforts, developing modular training manuals and certification programs for non-specialist workers, and developing public–private partnerships to build-up workforce capacity. Naslund and colleagues (2019) suggest that digital technologies can be tailored and used to support workforce development for non-specialist mental health workforce [49]. Digital platforms can support provider training, diagnostic tools, treatment guidance, and clinical or practice supervision. However, there is some evidence that combined digital and in-person training can have positive synergistic effects [50]. Additionally, future research is needed to investigate the optimal pedagogical training approaches required to support the non-specialist workforce, such as competency-based versus traditional knowledge acquisition approaches.

### Limitations

Findings should be interpreted in consideration of limitations. First, the concept mapping activity was introduced and the focal statement and initial brainstorming were generated during an IRT video call. Subsequent concept mapping activities were conducted asynchronously using an online platform and resulted in reduced participation. Several outreach attempts were made to solicit IRT member participation, but the study team learned that IRT members were fatigued by the platform and activities. It is possible that additional important and potentially changeable barriers and facilitators to non-specialist workforce training may have been identified if concept mapping activities continued in virtual or in-person meetings with greater participation. However, additional meetings would also likely have increased participant response burden. Second, almost 50% of statements were mapped to the Go-Zone, which could indicate that participants differed in their understanding of the rating process. All concept mapping instructions were reviewed by multiple team members working in the US and Kenya to ensure ease of interpretation, but language and technology barriers could have influenced participant behavior.

## Conclusion

The present study illustrates that training, development, and sustaining a non-specialist workforce for mental health care can’t be done in isolation, but rather will require the collaboration and engagement of policymakers, funders, mental health experts, the best research evidence, and the will to improve the lives of those living with depression and PTSD. In this study concept mapping activities provided a community-engaged approach for investigating how to impactfully grow and sustain the non-specialist mental health workforce for depression and PTSD service delivery. Participants rated many of the outer and inner context determinants and needed implementation strategies as both important and changeable, but structured concept mapping activities revealed that government action and resource allocation was perceived as the most critical and changeable approach to developing a sustainable mental health workforce. Integrating concept mapping with the EPIS framework aided in organizing results in a way that also advances implementation science. Combining this widely used framework with the mixed-method concept mapping approach helped reveal which factors and implementation strategies were perceived as optimal to address over implementation phases and which key stakeholders should be included in implementation efforts.

## Supplementary Information

Supplementary Material 1.

## Data Availability

De-identified concept mapping data may be available from the corresponding author on reasonable request.

## Abbreviations

EPIS: Exploration, Preparation, Implementation, Sustainment framework
IRT: Implementation resource team
LMICs: Low- and middle-income countries
PTSD: Post-traumatic stress disorder
SMART: Sequential, multiple, assignment randomized trial
SMART-DAPPER: Sequential, multiple, assignment randomized trial of two evidence-based treatments for depression and PTSD (the SMART-DAPPER study)
