# Supplementary material for: Multi-sector determinants of implementation and sustainment for non-specialist treatment of depression and post-traumatic stress disorder in Kenya: a concept mapping study
Source: Implement Sci Commun. 2025 May 7;6:55. doi: 10.1186/s43058-025-00744-7 (PMC12056999; doi:10.1186/s43058-025-00744-7)
Supplement: Supplementary file 1 — Supplementary Material 1. [file 43058_2025_744_MOESM1_ESM.docx]

**Supplemental Material.** Cluster importance and changeability ratings

| **Clusters and Statements** *(statement numbers in parentheses)* | **Importance** | **Changeability** |
| --- | --- | --- |
| **Cluster 1: Current workforce characteristics**  (1) High staff turnover negatively affects such collaborative efforts.  (18) Lack of clear roles and responsibilities.  (20) Resistance from professionals who see task shifting as poor medicine for poor people.  (21) High turn-over of trained workers.  (47) Unmotivated workforce.  (65) Do not introduce a new mental health cadre. | 3.5493 | 3.681633 |
| **Cluster 2: Exploration considerations for outer context engagement**  (3) Lack of ownership and responsibility from the Ministry of Health officials.  (17) Limited knowledge about mental illness prevents individuals from recognizing mental illness and seeking treatment.  (31) Ministry of Health view that mental health non-specialist workforce are not a reliable  cadre of personnel.  (45) Quality of health services. | 4.063825 | 3.99125 |
| **Cluster 3: Preparation considerations for the scope of what researchers can do**  (3) Lack of ownership and responsibility from the Ministry of Health officials.  (17) Limited knowledge about mental illness prevents individuals from recognizing mental illness and seeking treatment.  (31) Ministry of Health view that mental health non-specialist workforce are not a reliable cadre of personnel.  (45) Quality of health services. | 3.564333 | 3.7672 |
| **Cluster 4: Sustainment considerations - outer context issues**  (24) Insufficient participant reimbursement rates.  (52) Sustainment challenges.  (48) Government jobs are sought after.  (50) Consider stakeholder turnover during implementation. | 3.409975 | 3.568925 |
| **Cluster 5: Inner context implementation processes and tools**  (2) Provision of electronic navigation.  (4) Timely communication, both formal and informal.  (14) Provide in-person navigation.  (39) Addition to EMS.  (7) Effective treatment of mentally ill patient.  (15) Follow-up with participants at the community level to better understand access to care issues.  (58) Supervision for non-specialized providers. | 4.171971 | 4.178457 |
| **Cluster 6: Local capacity and partnerships**  (6) Community mental health service seeking.  (68) Ministry supported community health workers.  (69) Have a village health team (local people within the community).  (27) Identify partners with shared interests.  (40) Initiate sensitization sessions with stakeholders about mental health service delivery. | 4.35746 | 4.21008 |
| **Cluster 7: Financing for community health teams**  (10) Political will/support.  (41) Allocated financial resources (budgeted funds for this scale-up process).  (53) Payment for village health teams.  (34) Facilitate airtime reimbursement.  (57) Engage village health team members to provide care. | 4.07076 | 4.1554 |
| **Cluster 8: Outer context resource allocation/policy into action**  (5) Timely mental health assessment and treatment.  (8) Availability of drugs, equipment and commodity needed for mental health treatment.  (9) Availability of medicines.  (16) Survey questions should yield appropriate information about participant needs.  (35) Availability of mental health services in all levels of healthcare delivery points.  (36) Government and leadership policies that promote sustainability.  (30) Engage government (e.g., Ministry of Health, Ministry of Education, etc.) leadership in initial planning activities.  (46) The county's commitment to improving mental healthcare.  (56) Add mental health education to the village health team manual.  (66) Use existing community health strategy. | 4.46203 | 4.37367 |
| **Cluster 9: Workforce characteristics to enhance during implementation**  (11) Workforce capacity - staffing.  (12) Workforce qualifications, skill level.  (32) Non-specialist workforce should play a major role. | 4.070733 | 4.044867 |
| **Cluster 10: Workforce implementation strategies**  (42) Capacity building of healthcare workers at all levels in management of mental health.  (63) Engage professionals from different fields as non-specialist providers.  (44) Offering continuous medical education to the providers.  (59) Train psychologists to supervise community health workers.  (61) Train nurses and clinical officers on IPT.  (70) Involve educational institutions. | 4.263883 | 4.33345 |
| **Cluster 11: Cross-level workforce strategies**  (13) Collaboration between the Ministry of Health and mental health non-specialist workforce in implementing training program.  (37) Government and leadership policies that promote continuous mentorship/training for the mental health non-specialist workforce.  (38) Collaborative, public-private partnerships to build capacity for non-specialist workforce.  (49) Identify who will be trained (e.g., police, teachers).  (60) Have a targeted mental health curriculum.  (64) Make the existing mental health cadre more competent.  (19) Clear definition for identifying the mental health non-specialist workforce.  (33) Availability of human capital in the health products and technology field.  (67) Integrate non-specialists into government service system.  (71) Non-specialists help address stigma. | 4.30508 | 4.28123 |
| **Cluster 12: Training and education recommendations**  (28) Enhance psychoeducation.  (43) Expansion of mental healthcare from hospital to learning institutions.  (54) Training manual should be brief.  (55) Training manual should be modular.  (62) Provide a certificate for IPT. | 4.02628 | 4.33616 |
